# Defeating Bacterial Resistance and Preventing Mammalian Cells Toxicity Through Rational Design of Antibiotic-Functionalized Nanoparticles

**DOI:** 10.1038/s41598-017-01209-1

**Published:** 2017-05-02

**Authors:** Jessica Fernanda Affonso de Oliveira, Ângela Saito, Ariadne Tuckmantel Bido, Jörg Kobarg, Hubert Karl Stassen, Mateus Borba Cardoso

**Affiliations:** 1 Laboratório Nacional de Luz Síncrotron (LNLS)/Laboratório Nacional de Nanotecnologia (LNNano), Centro Nacional de Pesquisa em Energia e Materiais (CNPEM), CEP 13083-970, Caixa Postal 6192, Campinas, SP Brazil; 2 Instituto de Química (IQ), Universidade Estadual de Campinas (UNICAMP), CEP 13083-970, Caixa Postal 6154, Campinas, SP Brazil; 3 Laboratório Nacional de Biociências (LNBio), Centro Nacional de Pesquisa em Energia e Materiais (CNPEM), CEP 13083-970, Caixa Postal 6192, Campinas, SP Brazil; 4 Departamento de Bioquímica-Programa de Pós-graduação em Biologia Funcional e Molecular, Instituto de Biologia (IB), Universidade Estadual de Campinas (UNICAMP), CEP 13083-970, Caixa Postal 6109, Campinas, SP Brazil; 5 Faculdade de Ciências Farmacêuticas, Universidade Estadual de Campinas (UNICAMP), CEP 13083-871, Caixa Postal 6029, Campinas, SP Brazil; 6 Instituto de Química, Universidade Federal do Rio Grande do Sul (UFRGS), CEP 91501-970, Caixa Postal 15003, Porto Alegre, RS Brazil

## Abstract

The rational synthesis of alternative materials is highly demanding due to the outbreak of infectious diseases and resistance to antibiotics. Herein, we report a tailored nanoantibiotic synthesis protocol where the antibiotic binding was optimized on the silver-silica core-shell nanoparticles surface to maximize biological responses. The obtained silver nanoparticles coated with mesoporous silica functionalized with ampicillin presented remarkable antimicrobial effects against susceptible and antibiotic-resistant *Escherichia coli*. In addition, these structures were not cell-death inducers and different steps of the mitotic cell cycle (prophase, anaphase and metaphase) were clearly identified. The superior biological results were attributed to a proper and tailored synthesis strategy.

## Introduction

Since the discovery of penicillin, β-lactam antibiotics have been widely employed as antibacterial agents due to their broad spectrum of action and low toxicity^1^. However, the indiscriminate use of antibiotics resulted in the bacteria adaptation through the development of resistance^2^. Thus, pharmaceutical companies and researchers have devoted efforts to find out compounds free of resistance^3, 4^. Nevertheless, the methods/strategies adopted until now have not been efficient and new alternatives are being studied to prevent bacterial resistance^5^. Therefore, nanomaterials have been used as antimicrobial agents due to their unique physical and chemical properties^6^. Among many materials, silver nanoparticles (AgNPs) have shown potential biomedical applications, due to their antibacterial properties associated with slight propensity to induce microbial resistance and low toxicity for human cells^3, 7, 8^.

On the other hand, metallic nanoparticles have low colloidal stability and tend to aggregate in the biological environment. Thus, silica-coated metal nanoparticles have been considered as an efficient way to solve this problem. Moreover, silica coating allows further surface modification increasing biocompatibility of these materials^9^. Many different functional groups have been used to functionalize silica nanoparticles surfaces^10^. Functionalization with amine (-NH_2_) groups is very interesting since they can be used for further nucleophilic substitution^11^ forming amide bonds through carbodiimide reactions. In synthetic organic chemistry, compounds containing the carbodiimide functionality are dehydration agents and are often used to activate carboxylic acids towards amide or ester formation. Thus, amine functionalized nanoparticles can react with carboxylic acid from antibiotics.

Also, since 1950s, researchers are attempting to synthesize engineered materials in order to improve their biological response^12^. However, the rational design of biologically active nano-objects is still a challenge since the most helpful technique for this purpose is the molecular dynamic (MD) simulation. MD simulations are suitable for quantitative interaction measurements at the molecular level and, consequently, are able to predict the biological behavior of drugs. In parallel, molecular dynamics can also provide some understanding about thermodynamic and kinetic processes related to the permeability of drugs through lipid bilayers^13^. However, there is an enormous gap between the molecular simulations prediction and their possible use with nanoparticles since nanoparticles are difficult to simulate due to their large size considering the calculations used on MD.

Here we report a tailored nanoantibiotic synthesis protocol based on antibiotic binding optimization on the silver-silica core-shell surface. Bactericidal activity experiments were carried out using susceptible and antibiotic-resistant *Escherichia coli* to highlight the microbial activity of the synthesized system. Complementary, cytotoxicity assays were performed using HEK293T cells to exclude any residual toxicity effect of these materials for human cells.

## Results

**Figure 1:**
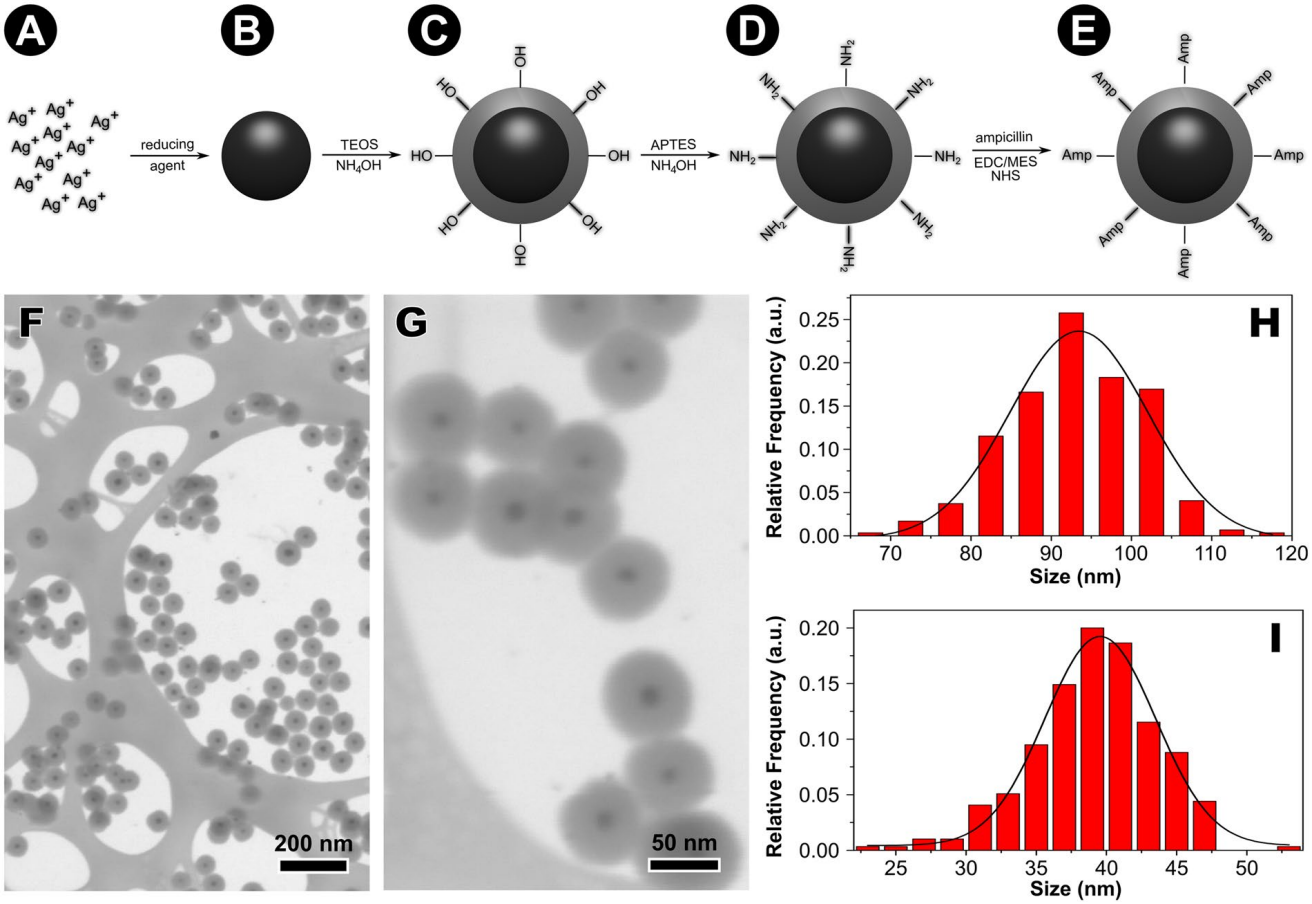
Synthesis steps to obtain silver nanoparticles functionalized with ampicillin. AgNPs (**B**) were formed by the reduction reaction of silver nitrate (**A**) in the presence of a reducing agent (the stabilizer is not shown for simplicity). Then a silica shell was produced by hydrolysis and condensation of tetraethyl orthosilicate (TEOS) and the produced core-shell Ag@SiO_2_ system is shown in (**C**). These nanoparticles are then coated with a thin silica layer (made of 3-aminopropyl triethoxysilane - APTES) to give a core-shell system with amino groups located on the surface of the nanoparticle (**D**). Finally, the amino-functionalized nanoparticles react with ampicillin to form the structure schematized in (**E**). This scheme is merely illustrative and is not in proportion to the actual size of the system. Transmission electron microscopy images of Ag@SiO_2_ nanoparticles obtained at (**F**) low and (**G**) high magnification. Core-shell (**H**) and shell thickne﻿s﻿s (**I**) size distributions obtained from TEM images.

The general nanoparticles synthesis scheme is presented in Fig. 1. First of all, silver nanoparticles (Fig. 1B) were formed by the reduction of silver ions (Fig. 1A) in the presence of a reducing agent. Next, a core-shell Ag@SiO_2_ system (Fig. 1C) was produced by tetraethyl orthosilicate hydrolysis and condensation in the presence of silver nanoparticles seeds. Then, Ag@SiO_2_ nanoparticles were coated with a thin silica/amine layer (Fig. 1D) that were further reacted with ampicillin (Fig. 1E).

AgNPs characterization details are presented in the Supplementary Information (Figure S1). The average AgNPs radii size was 12 nm as determined by SAXS. Silica shell was added to AgNPs through seeded polymerization technique by sol-gel reaction^14^ and Fig. 1F and G show TEM images of the synthesized nanoparticles. It is possible to observe that silver nanoparticles are completely coated by silica forming fairly regular spherical structures with considerably low polydispersity. Figure 1H and I show core-shell and shell thickness size distributions obtained from TEM images. The average size determined by TEM for the shell thickness is 39 nm while the core-shell structures have average size of 93 nm. Size distribution for Ag@SiO_2_ was also obtained by SAXS, which is a technique that prevents any possible experimental aggregation artifact due to the drying process (Figure S2B **–** Supplementary Information) and the results are in agreement with TEM images.

**Figure 2:**
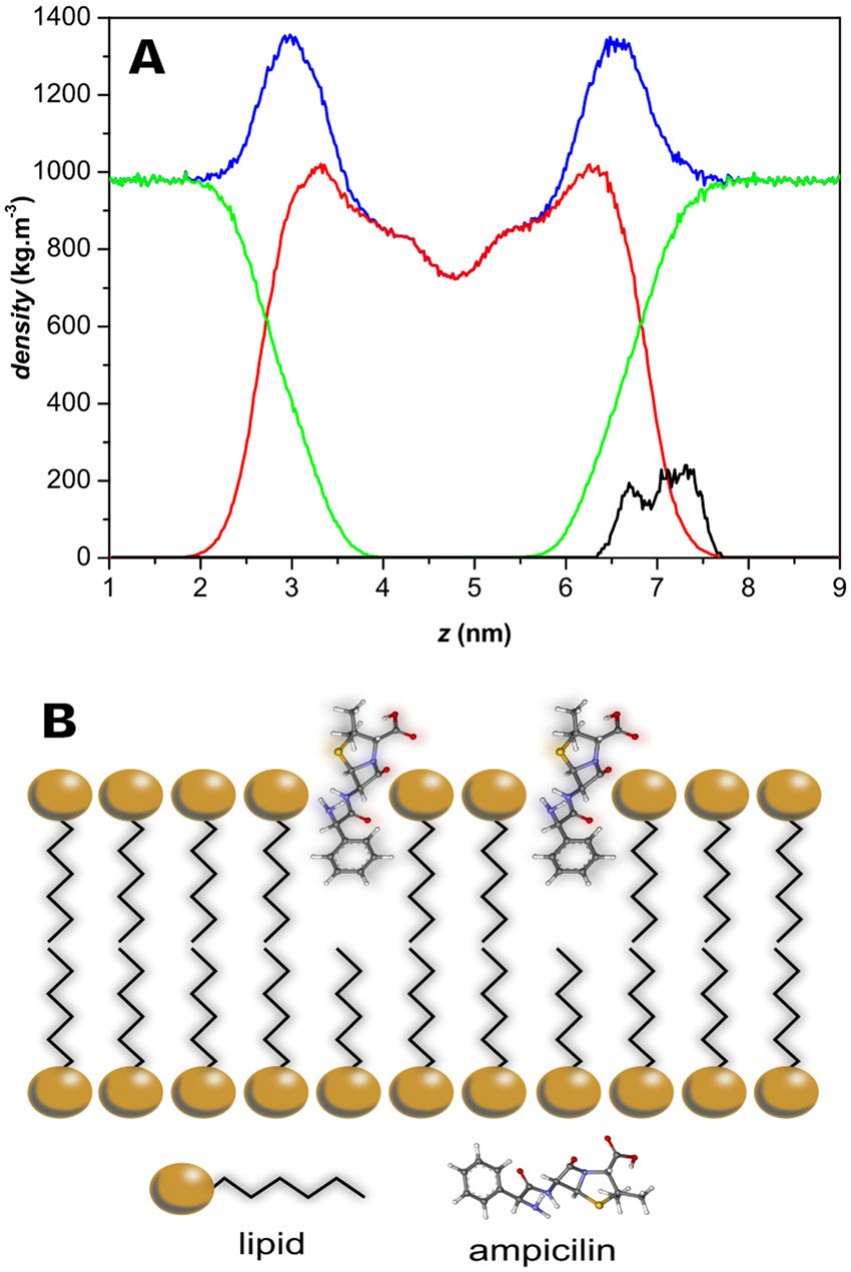
(**A**) Mass density profile (dp) of the simulated system (blue) between 190 and 200 ns and the contributions from the POPC molecules (red), the water phase (green) and the ampicillin molecules (black; multiplied by 10 to enhance visualization). (**B**) Schematic representation of ampicillin molecule and bacterial membrane interaction. This scheme is merely illustrative and is not in proportion to the actual size of the system.

Molecular dynamics simulation was performed to find the proper orientation in which the antibiotic is anchored in model membranes. This preferential binding orientation was used in order to optimize Ag@SiO_2_ nanoparticles surface functionalization with ampicillin. The density profile of the bilayer system, presented in Fig. 2A, reveals that the ampicillin molecules were inserted into the bilayer at the upper interface remaining within the hydrated head group region of the membrane. The obtained configuration exhibit orientations of the ampicillin molecules exposing the carboxyl group towards the hydration layer and turning the apolar phenyl group into direction of the lipophilic part of the membrane (Fig. 2B). The MD findings indicate that ampicillin is able to facilitate the approximation of the nanoparticle towards a membrane, especially, if we consider that ampicillin’s carboxyl group is less polar, when chemically bound to the nanoparticle, than in the simulated model system. Figure S3 of Supplementary Information presents the minimum distance between any atom of the ampicillin molecules and any atom of the POPC (2-oleoyl-1-palmitoyl-*sn*-glycero-3-phosphocholine) bilayer during the simulations as well as the radial pair distribution functions (*g*(*r*)).

**Figure 3:**
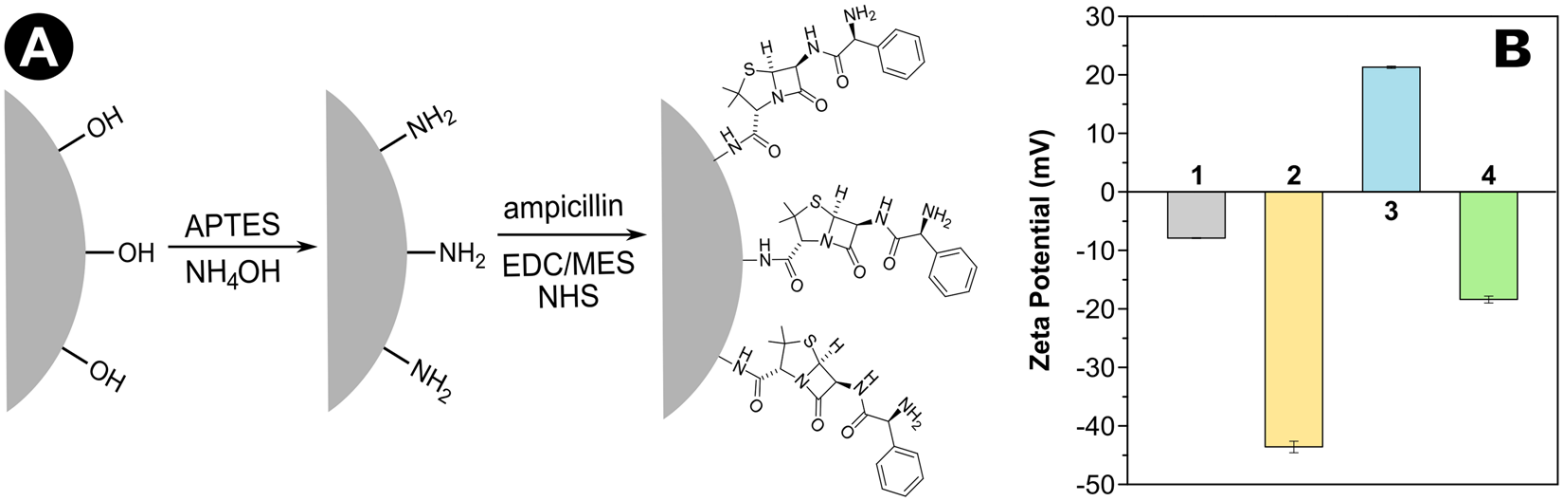
(**A**) Reaction steps to obtain nanoparticles functionalized with ampicillin. First, nanoparticles are coated with amino groups on the surface and the amino-functionalized nanoparticles react with the carboxyl groups of ampicillin through an acid-base coupling reaction to form Ag@SiO_2_-Ampicillin. This scheme is merely illustrative and is not in proportion to the actual size of the system. (**B**) Zeta potentials for (1) AgNP, (2) Ag@SiO_2_, (3) Ag@SiO_2_-NH_2_ and (4) Ag@SiO_2_-Ampicillin.

Thus, based on MD results, Ag@SiO_2_ was initially functionalized with amine group (-NH_2_) which was later reacted with carboxyl group (-COOH) from ampicillin according to the scheme shown in Fig. 3A. The synthesis mechanism is shown in Figure S4 of Supplementary Information while FT-IR spectra for Ag@SiO_2_, Ag@SiO_2_-NH_2_ and Ag@SiO_2_-Ampicillin, presented in Figures S5A, indicate the functionalization success. Complementary to FT-IR technique, zeta potential measurements were done and Fig. 3B shows the zeta potential values for the synthesized nanoparticles. AgNPs presented a slightly negative zeta potential and it becomes more negative after SiO_2_ coating, since –OH groups of silica surface are negatively charged in aqueous solution^15^. After surface functionalization, the zeta potential value becomes more positive and can be explained by the presence of amine groups (positively charged) on the silica surface. After the reaction with ampicillin, the zeta potential of the nanoparticles shows negative value again, since ampicillin is also negatively charged. Ampicillin mass fraction was determined by thermal decomposition using TGA technique (Figure S5B). For Ag@SiO_2_-NH_2_ the weight loss in the region from 120 to 600 °C is about 8.1 ± 0.6%. In the same region, the weight loss for Ag@SiO_2_-Ampicillin (blue line, Figure S5B) is 11.3 ± 0.6%. Taking into account that Ag@SiO_2_-NH_2_ was used as precursor for the Ag@SiO_2_-Ampicillin synthesis, the difference of 3.2 ± 0.6% between the two nanoparticles is due to the presence of ampicillin in the material. This value as well as the silver mass of each nanoparticle were used to normalize biological experiments presented below.

**Figure 4:**
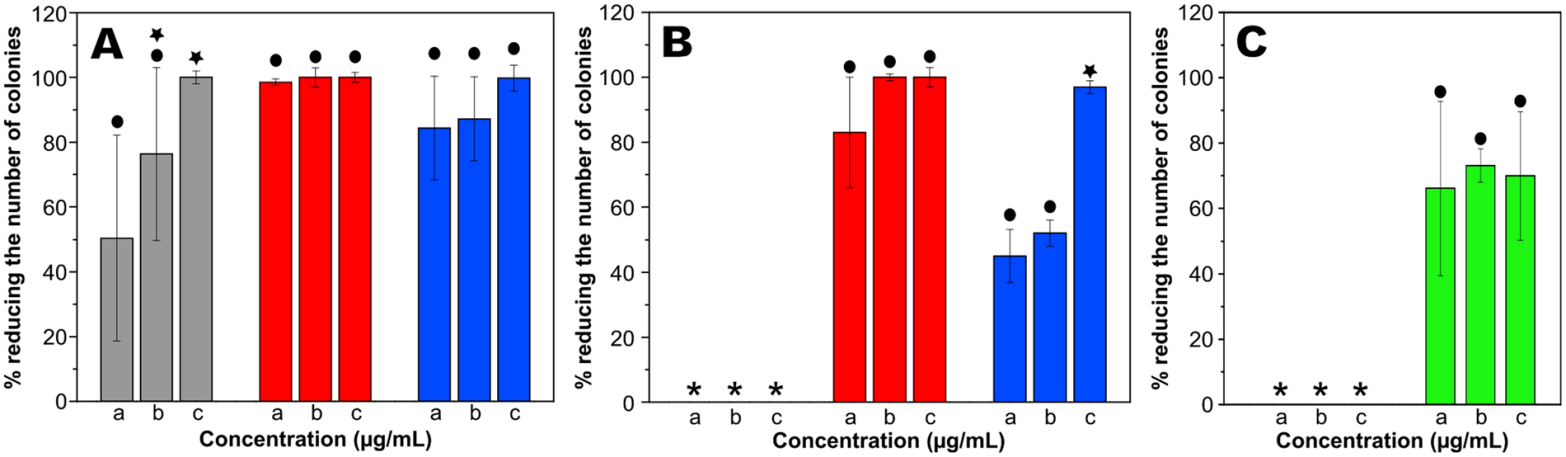
Comparative graph of the bactericidal effect of the synthesized material for (**A**) susceptible and (**B**) ampicillin-resistant *E*. *coli* bacteria. Ampicillin is represented by grey bars, Ag@SiO_2_ by red bars and Ag@SiO_2_-Ampicillin nanoparticles by blue bars. Based on TGA analysis, it was possible to determine the ampicillin content in Ag@SiO_2_-Ampicillin nanoparticles, so the concentrations a, b and c of ampicillin are 0.15, 1.55 and 7.76 µg/mL, respectively. For Ag@SiO_2_ and Ag@SiO_2_-Ampicillin, the concentrations a, b and c are 7.43, 74.30 and 372 µg/mL, respectively. (**C**) Comparative graph of the bactericidal effect of SiO_2_ and SiO_2_-Ampicillin nanoparticles for susceptible *E*. *coli* bacteria (SiO_2_-Ampicillin nanoparticles is represented by light green bars). The concentrations a, b and c of SiO_2_ and SiO_2_-Ampicillin are 7.33, 73.30 and 367 µg/mL, respectively. Additional information about the samples’ composition are presented in the Supplementary Information. The results shown are the average of each condition. The symbol * represents the concentrations that caused no inhibition of bacterial growth. Statistical analysis was performed according to the *t-test*. The symbols on top of the bars (circles and stars) indicate mean P < 0.05 for the same group. Symbols in common denote that the data are not statistically different. In addition, no statistical differences were observed when concentrations a, b or c of Ag@SiO_2_ and Ag@SiO_2_-Ampicillin were compared (panel A). In panel B, significant statistical differences were found for concentrations a and b while no differences were verified for concentration c when Ag@SiO_2_ and Ag@SiO_2_-Ampicillin were compared.

The synthesized materials were tested in cultures of susceptible and ampicillin-resistant *Escherichia coli* and Fig. 4 shows a comparison of the reduction in the number of colonies due to the materials’ application. Comparison was done taking into account the control (bacteria free of nanoparticles) to calculate the percentage of reduction in the number of colonies. As expected, the bacterial colony growth was reduced in a concentration dependent manner.

Ampicillin is a well-known antibiotic that inhibits bacterial cell wall synthesis by binding to peptidoglycan-synthesizing enzymes^16^. In fact, the tests carried out with ampicillin (grey columns, Fig. 4A) show that *E*. *coli* is susceptible to this antibiotic, even at low concentrations (0.15 µg/mL). Once ampicillin-resistant *E*. *coli* presents the plasmid pET-32a(+), which confers resistance to ampicillin, the antibiotic was not able to decrease bacterial growth at any of the studied concentrations (Fig. 4B).

In the presence of Ag@SiO_2_ (red columns, Fig. 4), a 100% inhibition of bacterial growth (except for the most diluted solution) was seen for both bacteria. Such effectiveness of this material may be mainly attributed to the Ag^+^ ions release effect from silver core through the porous silica layer, producing and maintaining its bactericidal effects^17^.

The results for the bacterial inhibition growth caused by Ag@SiO_2_-Ampicillin (blue columns, Fig. 4) are similar to the ones obtained with core-shell system (red columns) for the most concentrated solutions. However, it was observed a reduction in the number of colonies of about 80% in the lowest concentration for *E*. *coli* (blue columns, Fig. 4A) and about 50% for ampicillin-resistant *E*. *coli* (blue columns, Fig. 4B). Comparatively, it was not expected such decrease in inhibitory properties of Ag@SiO_2_-Ampicillin at low concentrations if compared to Ag@SiO_2_. However, the nanoparticles’ functionalization occurs through the formation of an amide bond between COO^−^ group from ampicillin and the NH_2_^+^ present on the silica surface. Therefore, the smaller effectivity of Ag@SiO_2_-Ampicillin when compared to Ag@SiO_2_ cannot be attributed to the antibiotic binding. One possible and reasonable explanation for this result may be related to the fact that chemical reactions during surface functionalization (schematically shown in Fig. 3A) caused a partial obstruction of the pores, preventing the leaching of ionic silver. The schematic illustration of material clogged pores after functionalization is presented in Figure S6 of the Supplementary Information. This partial obstruction interpretation is based on the drastic decrease of the surface area from 32 m^2^/g (Ag@SiO_2_) to 13 m^2^/g (Ag@SiO_2_-Ampicillin) after functionalization, according to the data obtained by nitrogen adsorption/desorption analysis. Within this scenario, we suggest that the leaching of silver ions is more pronounced in Ag@SiO_2_ than in Ag@SiO_2_-Ampicillin resulting in distinct biocidal activities. Then, it is very complex to deconvolute the bactericidal contribution related to the silver ions and the one coming from the grafting of ampicillin. In order to validate the bactericidal efficacy of ampicillin functionalization, two distinct silica nanoparticles were synthesized in absence of AgNP cores. These two particles were synthesized aiming to have an average size similar to Ag@SiO_2_-Ampicillin. Details about these two samples are provided in the Supplementary Information. The difference between these samples is only attributed to the ampicillin functionalization since one is made of bare silica (SiO_2_) while the other is the same silica structure functionalized with ampicillin (SiO_2_-Ampicillin). Now, the possible effect of silver ions is totally discarded and the only effect observed is related to the ampicillin grafting. Figure 4C clearly shows that SiO_2_-Ampicillin presents a much higher bactericidal power if compared to SiO_2_ highlighting that ampicillin functionalization is an effective way to increase the biological activity. Based on this result, we suggest here that ampicillin is likely to facilitate the nanoparticle anchoring on the outermost bacteria membrane. The anchoring probably results in nanoparticles’ penetration into bacteria membrane and causes the microorganism death.

The possible cytotoxic effect of the two most effective nanoparticles against bacteria was investigated using HEK293T cells. Figure S7 of Supplementary Information shows cellular viability of HEK293T cells treated with 372 µg/mL of Ag@SiO_2_ or Ag@SiO_2_-Ampicillin (highest concentration used during bactericidal tests). Thapsigargin (30 µM) was used as reference control drug, which effectively induces cell death by inhibiting Ca^2+^ ATPases^18^, and the measurements were carried after 24 and 48 h of incubation. We observed that thapsigargin caused a reduction in cell viability to 4% during 24 h of treatment, whereas practically 100% of the cells were dead after 48 h. Ag@SiO_2_ system showed a strong cytotoxic effect for both treatment periods, reducing the cell viability to around 20% after 48 h of incubation. On the other hand, Ag@SiO_2_-Ampicillin showed promising results when compared to Ag@SiO_2_ system since no significant viability reduction was observed along the time period studied.

**Figure 5:**
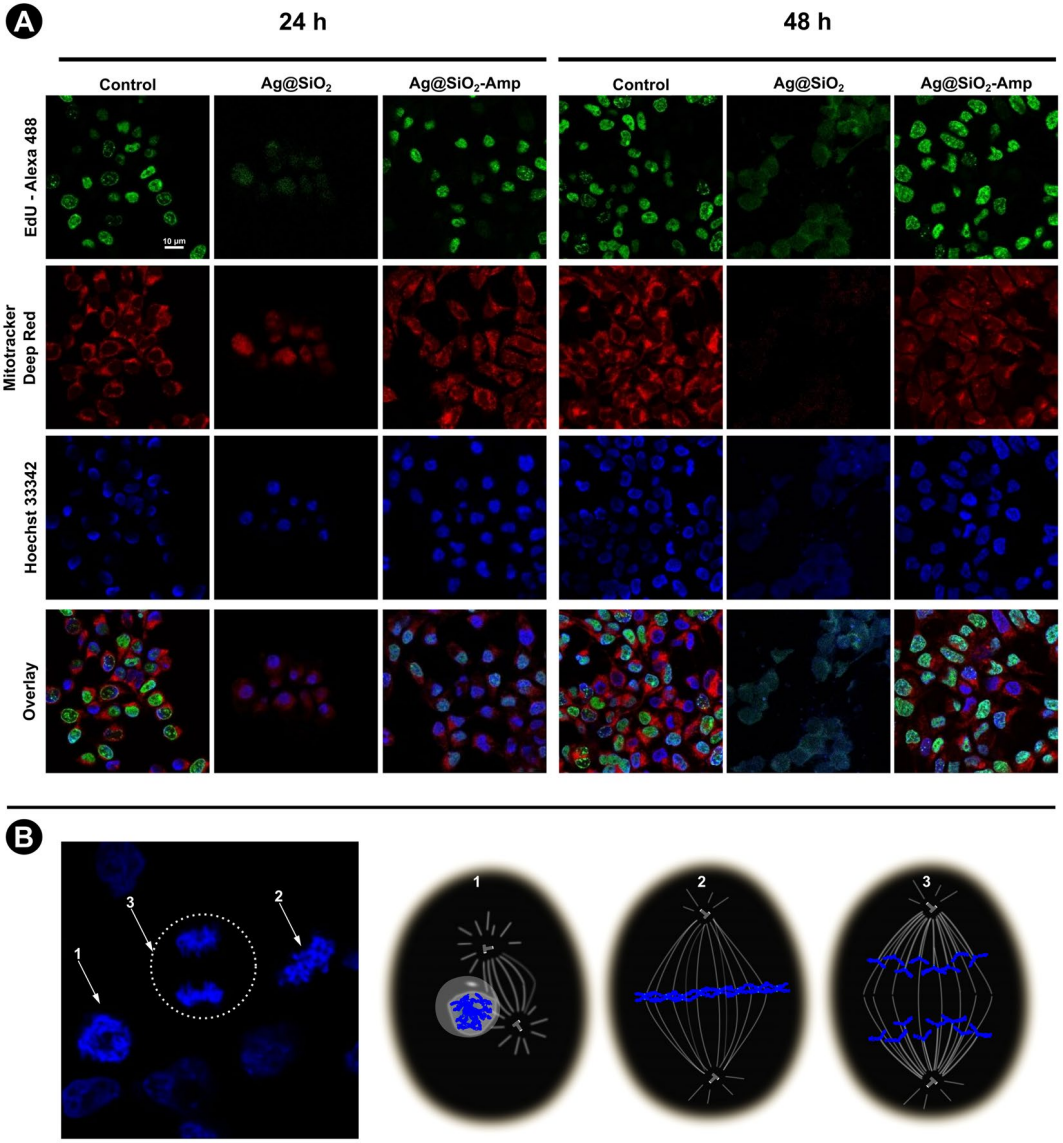
Cells images during the cytotoxicity tests. (**A**) First column in 24 and 48 h treatment corresponds to the control test in absence of nanoparticles, second column corresponds to the cells in the presence of the Ag@SiO_2_ and third column corresponds to the cells in the presence of the Ag@SiO_2_-Ampicillin. EdU-Alexa 488 line represents proliferating cells; MitoTracker^®^ Deep Red line indicates mitochondria in the cytoplasm; Hoechst 33342 line represents the cell nuclei and the last line represents the overlay of the three images for each condition. (**B**) Mitoses phases observed in confocal images. White arrows in confocal image and schemes 1, 2 and 3 represent prophase, metaphase and anaphase, respectively, after 48 h of Ag@SiO_2_-Ampicillin treatment. The schemes are merely illustrative for the phases observed and are not in proportion to the actual size of the system.

Confocal microscopy was used to identify HEK293T structures by specific labeling of subcellular organelles to better understand and evaluate the results from cell viability tests. Figure 5 shows images of HEK293T cells in the absence (control sample) and in the presence of the Ag@SiO_2_ or Ag@SiO_2_-Ampicillin after 24 and 48 h of incubation.

HEK293T cells were labeled with EdU-Alexa488, MitoTracker^®^ Deep Red and Hoechst 33342. EdU is a thymidine analogue in which a terminal alkyne group replaces the methyl group in position 5 leaving to its incorporation into cellular DNA during the S (Synthesis) phase of Interphase, when DNA is replicated^19^. MitoTracker^®^ Deep Red stains viable mitochondria in the cytoplasm. Hoechst dyes are often used as substitutes for another nucleic acid stain called DAPI^20^, since they are less toxic. Furthermore, the additional ethyl group of the Hoechst 33342 dye renders it more cell permeable, thereby allowing live cell staining without the necessity of previous fixation.

Under control conditions (absence of nanoparticles) and after Ag@SiO_2_-Ampicillin treatment, most cells incorporated EdU indicating regular cell proliferation. Normal nucleus in blue and mitochondrial staining in red were also observed under these conditions. However, in the presence of Ag@SiO_2_ nanoparticles, cells presented drastic phenotypic changes. After incubation for 24 h, EdU-Alexa488 staining was weak and diffused throughout the cell and mitochondrial signal was also dispersed in the cytoplasm and nucleus. After treatment for 48 h, mitochondrial signal was lost and Hoechst 33342 staining was dispersed throughout the cytoplasm, which indicates cellular death. Thus, we can infer that Ag@SiO_2_ is cytotoxic to HEK293T cells in the conditions studied here, which corroborates with the MTS assay. On the other hand, no signal for apparent cytotoxicity was observed in the presence of Ag@SiO_2_-Ampicillin. In addition, Fig. 5B shows images of mitosis phases of Ag@SiO_2_-Ampicillin-treated HEK293T cells. It is possible to observe cells during three mitosis phases: prophase (arrow and scheme 1, Fig. 5B), ﻿metaphase﻿ (arrow and scheme 2, Fig. 5B) and anaphase (arrow and scheme 3, Fig. 5B). These results suggest that, for at least 48 h, almost no toxicity or cell growth inhibition was observed in the presence of Ag@SiO_2_-Ampicillin and that the antibiotic probably acts as toxic-protective organic molecule. Thus, nanoparticles coated with ampicillin were not able to interfere during the cellular metabolism since different mitosis cell phases were seen in the presence of Ag@SiO_2_-Ampicillin.

The results presented here show that Ag@SiO_2_-Ampicillin nanoparticles have no significant toxicity to mammalian cells with efficient antibacterial properties against susceptible and antibiotic-resistant bacteria. A possible explanation can be related to the action mechanism in bacteria. Usually, antibiotics permeate and/or affect bacteria cell wall whereas mammalian cells are maintained. These two cell membranes have different compositions, and normally the hydrolysis of bonds between the saccharides units of peptidoglycan skeleton of bacteria membrane is the death cause. It is well known that ampicillin inhibits enzymes present in the bacterial cell walls, such as transpeptidases and carboxypeptidases. These enzymes are essential for peptidoglycan layer synthesis of bacterial cell walls. According to the literature^21^, β-lactam antibiotics resemble d-alanylalanine peptide fragment, which the enzyme takes for its substrate while facilitates their binding to the active site of penicillin-binding proteins (PBPs). PBPs are a group of enzymes found anchored in the cell membrane, which are involved in the cross-linking of the bacterial cell wall. The antibiotic irreversibly binds (acylates) to the serine residue of the PBP active site, disrupting cell wall synthesis. A schematic representation of this process is shown in Figure S8. Since human cells do not synthesize or need peptidoglycans, antibiotics are not able to affect them^21^.

## Conclusions

We report here an antimicrobial strategy using silver nanoparticles coated with ampicillin-functionalized silica (Ag@SiO_2_-Amp) to defeat susceptible and antibiotic-resistant *E*. *coli* bacteria. Ag@SiO_2_-Amp presented similar bactericidal efficacy to its precursor (Ag@SiO_2_) for non-resistant bacteria while a subtle decrease was observed for the ampicillin-resistant microorganism. Further, the antibiotic-capped nanoparticles were not cytotoxic since the mitotic cell cycle was not affected by the presence of these structures while high cellular death levels were verified for Ag@SiO_2_. The approaches used here offer a promising strategy to design non-toxic functionalized nanoparticles which can be used in antimicrobial, antiviral or anti-cancer therapies.

## Experimental Procedure

### Materials

Silver nitrate (AgNO_3_), polyvinylpirrolidone (PVP 40000), tetraethyl orthosilicate (TEOS), (3-Aminopropyl)triethoxysilane (APTES), ampicillin, 2-(N-Morpholino) ethanosulfonic acid (MES), N-hydroxysuccinimide (NHS) and 1-ethyl-3-(3-dimethylaminopropyl)carbodiimide (EDC) were obtained from Sigma-Aldrich. Ethanol, ethylene glycol and ammonium hydroxide were purchased from Synth. All chemicals and reagents were used as received without further purification. Water used in all procedures was obtained from a water purification system (Purelab from ELGA) and had a measured resistivity of 18.2 MΩ cm^−1^.

### Silver nanoparticles synthesis

AgNPs were synthesized according to Graf *et al*.^14^ and Silvert *et al*.^22^. In summary, 1.5 g of PVP were completely dissolved in 75 mL of ethylene glycol (EG) under magnetic stirring at room temperature. Then, 0.05 g of AgNO_3_ was added to the solution and stirred until complete dissolution. Then, the solution was heated from 22 to 120 °C at a heating rate of 5 °C/min, keeping at this temperature for 1 hour. Finally, the system was cooled down to room temperature in a water bath with magnetic stirring.

### Silica shell formation around silver nanoparticles (Ag@SiO_2_)

The addition of the silica shell on AgNPs surface was performed using the seeded polymerization technique by sol-gel reaction described by Graf *et al*.^14^. Initially, the AgNPs previously described were centrifuged at 6000 rpm for 10 minutes in acetone, the supernatant was discarded and the pellet was redissolved in 50 mL of ethanol. Then, the solution was centrifuged again at 15000 rpm for 10 minutes. The precipitate was redissolved in 20 mL of ethanol and centrifuged again at 15000 rpm for 15 min. The precipitate was resuspended in an ammonia solution (4.2% [v/v] ethanol), and immediately a TEOS solution (10% [v/v] ethanol) was added to the mixture under stirring. The reaction was stirred overnight, followed by centrifugation at 8000 rpm for 10 min. The precipitate was washed with ethanol and then dried to obtain Ag@SiO_2_ composite.

### Synthesis of Ag@SiO_2_ functionalized with amine groups (Ag@SiO_2_-NH_2_)

The reaction with 3-aminopropyltriethoxysilane (APTES) was performed in two stages using the same reaction flask. Initially, it was performed the same procedure as previously described and, after overnight stirring, 10.5 µl of APTES was added to the core-shell solution. After APTES addition, the reaction was kept overnight under magnetic stirring, followed by centrifugation at 8000 rpm for 10 min. The precipitate was washed with ethanol and dried to obtain Ag@SiO_2_-NH_2_.

### Synthesis of Ag@SiO_2_ functionalized with ampicillin (Ag@SiO_2_-Ampicillin)

Under magnetic stirring, 56.16 µl of EDC were added to a NHS solution, followed by the immediate addition of ampicillin solution. Stirring was maintained for 1 hour, and then Ag@SiO_2_-NH_2_ in MES (0.1 M, pH 5) was added. The reaction was left overnight under magnetic stirring at room temperature, followed by centrifugation at 8000 rpm for 10 min. The precipitate was washed with ethanol and dried to obtain Ag@SiO_2_-Ampicillin composite.

### Nanoparticles characterization

Surface plasmon resonance of silver nanoparticles was investigated with Agilent 8453 equipment using UV-Vis quartz cuvette (10 mm optical length). Deionized water was recorded as reference before recording the absorbance spectrum of the samples at room temperature. All samples were diluted until the absorption maximum of 0.8 was reached since the optical density of the as-synthesized samples was excessively high for recording the spectrum.

Zeta potential measurements were performed at room temperature using Malvern Zetasizer – Nano ZS90 equipment where an electric field is applied to the solution resulting in the particles movement. The velocity associated to this movement is related to zeta potential which was measured using a laser technique called M3-PALS (Phase Analysis Light Scattering). It enables electrophoretic mobility calculation and, consequently, the particles zeta potential determination. All measurements were made in triplicate.

Small-angle X-ray scattering (SAXS) measurements were carried out in D1B-SAXS1 beamline at the LNLS in order to determine the size and polidispersity of the synthesized nanoparticles. The scattered X-ray beam presenting wavelength (λ) of 1.488 Å was detected on a Pilatus 300k detector. The sample-to-detector distance was 3050 mm, covering a scattering vector *q* $(q=\frac{4\pi }{\lambda }\,\sin \,\theta )$ ranging from 0.04 to 1.1 nm^−1^, where 2*θ* = scattering angle. All measurements were performed at room temperature and silver behenate was measured under the same conditions to calibrate the sample-to-detector distance, the detector tilt and the direct beam position. Transmission, dark current and mica sheet corrections were performed. The normalized scattering image of the samples was then subtracted from the normalized scattering image of pure water and the isotropic result was radially averaged to obtain the *I*(*q*) vs *q*. The absolute calibration was achieved by the use of water as a standard of known differential cross section (1.65 × 10^−2^ cm^−1^)^23^. Fitting procedures were carried out using the SASfit software.

Transmition electron microscopy (TEM) was used to investigate size and morphology of the synthesized nanoparticles. A drop of the suspension containing the nanoparticles was deposited on a grid of 400 meshs and it was examined using FEI Inspect F50 at the Brazilian Laboratory of Nanosciences (LNNano) operating at an accelerating voltage of 10–20 keV.

Thermogravimetric analyses (TGA) were performed to estimate the mass ratio between silica and ampicillin. TGA measurements were carried out with a *Perkin-Elmer Thermogravimetric Analyser Pyris 1 TGA* by heating from 20 to 1000 °C at a rate of 20 °C/min in air.

Nitrogen adsorption-desorption isotherms were measured on an Autosorb^®^-1 series instrument (Quantachrome, Boynton Beach, USA). The samples were degassed at 110 °C at relative pressure range of 0.07–0.30 P/P^0^. The surface area (*S*_BET_) was determined by the BET (Brunauer–Emmett–Teller) method.

Fourier transform infrared (FT-IR) spectra were recorded in a transmission mode on a Perkin Elmer FT-IR spectrophotometer (model Spectrum Two) using KBr pellets under ambient conditions. The pellets were subjected to 32 scans at a resolution of 4 cm^−1^.

### Molecular dynamics simulation

The zwitterionic form of the ampicillin molecule (anionic carboxylate group, protonated NH_2_-group) has been geometry-optimized at the B3LYP//6-311++ G(d,p) level using the Gaussian 98 software^24^. Afterwards, the electrostatic potential has been computed at the HF-6-31G(d) level and atomic point charges were extracted from the double stage RESP fitting procedure^25^. The molecular geometry and the atomic point charges have been utilized to define ampicillin’s topology for the AMBER99SB-ildn force field^26^. The missing angular deformation terms involving the four- and five-membered ring systems have been adapted from Stroganov *et al*.^27^.

The membrane is represented by a bilayer containing 128 molecules of 1-palmitoyl-2-oleoyl-sn-glycero-3-phosphocholine (POPC) described by the Stockholm lipid parameterization^28, 29^. The starting membrane has been obtained from lipidbook^30^. We have added additional water molecules of TIP3P type^31^ for the insertion procedure of four ampicillin molecules. The simulated system consisted of 128 POPC, 7687 water, and four ampicillin molecules.

The MD simulation has been performed with the GROMACS 4.5.5 package^32^ in the NpT ensemble with the temperature maintained at 310 K by velocity rescaling^33^ and the pressure fixed at 1 bar by the semi-isotropic Parrinello-Rahman barostat^34^. Constraining the bond lengths by the LINCS algorithm^35^ (SETTLE in the case of water molecules^36^), an integration time of 0.002 ps was employed. A spherical cutoff radius of 1.25 nm was used correcting long-range electrostatic interactions by the PME method^37^. The simulation has been extended to a total simulation time of 200 ns. After 160 ns, we observed a satisfactory convergence of properties such as box size, density, and intermolecular interaction energies. The computed area per lipid is 0.653 nm² which is in excellent agreement with other simulation studies on the POPC bilayer^28^. The last 10 ns of the simulation were used to compute structural properties of the system.

### Bactericidal susceptibility tests

Bacteriological tests were conducted with DH5α susceptible and ampicillin-resistant *Escherichia coli* strains. Both *E*. *coli* were provided by Prof. Dr. Dulce Helena Ferreira de Souza, from Chemistry Department, Federal University of São Carlos (DQ - UFSCar). Initially, *E*. *coli* strains were separately inoculated in a flask containing 5 mL of Luria Bertani (LB) broth (containing 10 g.L^−1^ of peptone, 10 g.L^−1^ of NaCl and 5 g.L^−1^ of yeast extract). The flask was stirred for 5 hours with an orbital shaker (200 rpm at 37 °C) and then 5 µL of these solutions were, separately, diluted with 50 mL of LB broth (Dilution 1). Then, 10 mL of Dilution 1 was diluted with 40 mL of LB broth (Dilution 2). A mixture containing 50 µL of Dilution 2, 1 mL of LB broth and 700 µL of aqueous solution of composites were stirred for 5 hours with an orbital shaker (200 rpm at 37 °C). In order to evaluate that the bacteria viability decrease is due to the tested materials, a growth control was prepared simultaneously. Thus, we were able to evaluate if the synthesized materials were capable of inhibit and kill the tested bacteria. For the growth control, 50 µL of Dilution 2, 1 mL of LB broth and 700 µL of sterile Mili-Q water was stirred for 5 hours with an orbital shaker (200 rpm at 37 °C). Then, 5–10 µL of these solutions were, separately, diluted with 1 mL of LB broth (Dilution 3). At this point, 100 µL of Dilution 3 were spread onto LB agar plates and incubated at 37 °C for 17 hours. The same procedure was followed for growth control. For ampicillin-resistant *E. coli*, it was necessary to add ampicillin solution (100 µg/mL) to LB liquid medium as well as into agar plates, in order to prevent the growth of susceptible bacteria. The bactericidal activity of synthesized composites was evaluated by counting the number of colonies formed on agar plates. All the experiments were conducted in triplicate, performed simultaneously and the average values were reported. Concentration of silver was used as normalization factor and the calculation is provided in the Supplementary Information. Statistical analyses were performed with the Students *t-test*.

### Cell culture

HEK293T cells were maintained in high glucose Dulbecco’s Modified Eagle Medium (DMEM, Sigma) supplemented with 10% fetal bovine serum (FBS, Gibco), 100 units/mL penicillin and 100 µg.mL^−1^ Streptomycin (Gibco). All cells were grown in 96-well plates at 37 °C with 5% CO_2_ atmosphere incubation.

### MTS cell viability assay

About 1 × 10^4^ cells per well were seeded onto 96-well plates. After 24 h, the media was replaced for a fresh one containing the nanoparticles. Then, after nanoparticle incubation for 24 or 48 h, cell viability assay was performed using CellTiter 96^®^ AQueous One Solution Cell Proliferation Assay Kit (Promega). The assay was performed by adding new freshly media containing 20 µL of Cell Titer 96^®^ AQueous One Solution into each culture well, followed by 1 h incubation at 37 °C and 5% CO_2_. Then, the absorbance was measured at 490 nm with a 96-well plate reader (Perkin Elmer EnSpire Multimode Reader) at the Brazilian Biosciences National Laboratory (LNBio, Campinas, Brazil). All experiments were conducted in quadruplicate, performed simultaneously and the average values were reported. The cell viability was expressed as a percentage relative to the control as calculated by Equation 1.

1 $$Cell\,viability( \% )=\frac{{{\rm{OD}}}_{{\rm{sample}}}}{O{D}_{Control}}\times 100$$

where *OD*_sample_ is the optical density of the sample in presence of the cells and *OD*_control_ is the optical density of the control group (untreated cells).

### Cell proliferation and viability

HEK293T cells were seeded onto glass coverslips in 24-well plates overnight and the media was replaced with fresh media containing 138 µg/mL Ag@SiO_2_ or Ag@SiO_2_-Ampicillin nanoparticles. After 24 and 48 h, 20 µM of 5-ethynyl-2′-deoxyuridine (EdU, Life Technologies) was incubated with new media for 3 hours at 37 °C and 5% CO_2_. In order to stain viable mitochondria, cells were also incubated with 200 nM MitoTracker Deep Red FM (Molecular Probes, Invitrogen) in Opti-MEM I Media (Life Technologies) for 30 minutes at 37 °C, 5% CO_2_, protected from light, followed by 10 minutes fixation at room temperature with 3.7% formaldehyde (v/v) diluted in PBS. A Cu(I)-catalyzed cycloaddition (“click” chemistry) was then performed with 10 µM AlexaFluor 488 azide (Life Technologies), 1 mM CuSO_4_ and 100 mM ascorbic acid diluted in PBS. The reaction was incubated for 1 h at 4 °C, protected from light^19^. Cells were washed once with PBS and nuclei were stained with Hoechst 33342. Slides were mounted using Fluoromount G. Analysis were carried out using excitation wavelength of 405 nm for Hoechst 33342, 633 nm for MitoTracker^®^ Deep Red FM and 488 nm for AlexaFluor 488 azide; emission wavelength range from 418 to 585 nm for Hoechst 33342, from 677 to 728 nm for MitoTracker^®^ Deep Red FM and from 501 to 544 nm for AlexaFluor 488 with an objective lens of 63×/1.4 oil. The cell images were obtained from Confocal Laser Scanning Microscope Leica TCS SP8 at the Brazilian Biosciences National Laboratory (LNBio, Campinas, Brazil).

## Electronic supplementary material

Defeating Bacterial Resistance and Preventing Mammalian Cells Toxicity Through Rational Design of Antibiotic-Functionalized Nanoparticles
