# Supplementary material for: Defeating Bacterial Resistance and Preventing Mammalian Cells Toxicity Through Rational Design of Antibiotic-Functionalized Nanoparticles
Source: Sci Rep. 2017 May 2;7:1326. doi: 10.1038/s41598-017-01209-1 (PMC5430956; doi:10.1038/s41598-017-01209-1)
Supplement: Supplementary file 1 — Defeating Bacterial Resistance and Preventing Mammalian Cells Toxicity Through Rational Design of Antibiotic-Functionalized Nanoparticles [file 41598_2017_1209_MOESM1_ESM.pdf]

Supplementary Information

# Defeating Bacterial Resistance and Preventing Mammalian Cells Toxicity Through Rational Design of Antibiotic-Functionalized Nanoparticles

Jessica Fernanda Affonso de Oliveira[1,2], Ângela Saito [3,4], Ariadne Tuckmantel Bido[1,2], Jörg Kobarg[4,5], Hubert Karl Stassen[6], Mateus Borba Cardoso[1,2*]

[1] Laboratório Nacional de Luz Síncrotron (LNLS) / Laboratório Nacional de Nanotecnologia (LNNano), CEP 13083-970, Caixa Postal 6192, Campinas, SP, Brazil.

[2] Instituto de Química (IQ), Universidade Estadual de Campinas (UNICAMP), CEP 13083-970, Caixa Postal 6154, Campinas, SP, Brazil.

[3] Laboratório Nacional de Biociências (LNBio), CEP 13083-970, Caixa Postal 6192, Campinas, SP, Brazil,

[4] Departamento de Bioquímica-Programa de Pós-graduação em Biologia Funcional e Molecular, Instituto de Biologia (IB), Universidade Estadual de Campinas (UNICAMP), CEP 13083-970, Caixa Postal 6154, Campinas, SP, Brazil.

[5] Faculdade de Ciências Farmacêuticas, Universidade Estadual de Campinas (UNICAMP), CEP 13083-970, Caixa Postal 6154, Campinas, SP, Brazil.

[6] Instituto de Química, Universidade Federal do Rio Grande do Sul (UFRGS), CEP 91501-970, Caixa Postal 15003, Porto Alegre, RS, Brazil.

* Corresponding author (M.B.C.)

E-mail: cardosomb@lnls.br and cardosomb@lnnano.cnpem.br

Fax: +55 19 3512 1004

Tel: +55 19 3512 1045

**Contents**

**1.** *Silver nanoparticles characterization*

**2.** *UV-Vis and SAXS patterns for Ag@$SiO_2$ nanoparticles*

**3.** *Molecular dynamics simulation*

**4.** *Reaction mechanism of amide bond formation*

**5.** *FT-IR spectra and TGA curve of synthesized nanoparticles*

**6.** *Bactericidal tests*

**7.** *Schematic representation of partial pores obstruction during functionalization*

**9.** *SAXS patterns, size distribution and SEM image for $SiO_2$ nanoparticles*

**9.** *Cytotoxicity assay*

**10.** *Penicillin-binding proteins (PBPs)*

**11.** *Determination of core-shell concentration, using Ag as normalization factor*

**12.** *Synthesis of silica nanoparticles ($SiO_2$)*

## *1. Silver nanoparticles characterization*

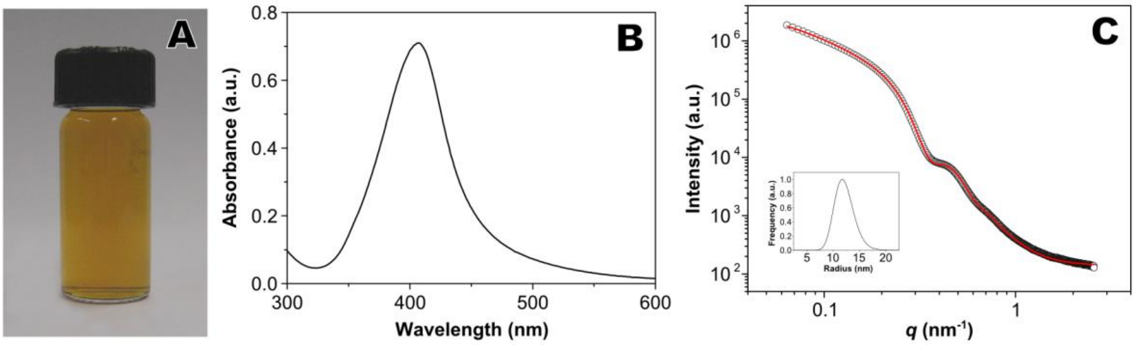


**Figure S1.** (A) Photograph image of the as-synthesized silver nanoparticles capped with PVP and (B) its corresponding UV-Vis spectrum. (C) SAXS pattern for silver nanoparticles sample (open circles) and its corresponding fit (solid line). Inset: Silver nanoparticles size distribution obtained from SAXS fit presented in Figure 1C.

## *2. UV-Vis and SAXS patterns for Ag@SiO₂ nanoparticles*

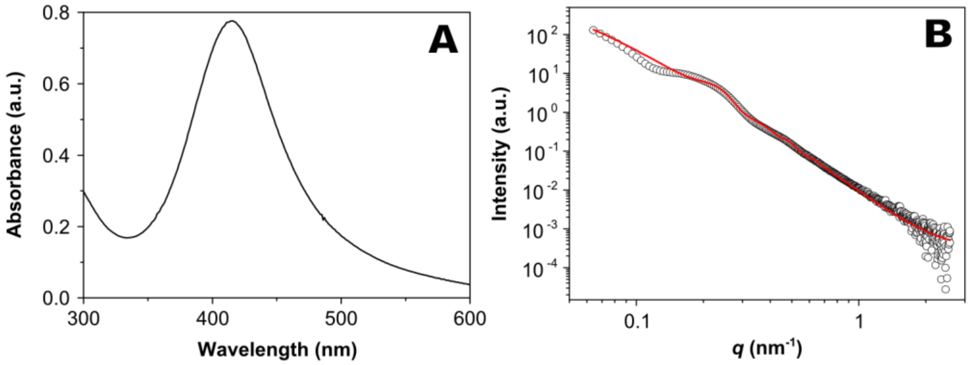


**Figure S2.** (A) UV-Vis spectrum and (B) SAXS pattern and its corresponding fit (solid line) for Ag@$SiO_2$ nanoparticles.

### *3. Molecular dynamics simulation*

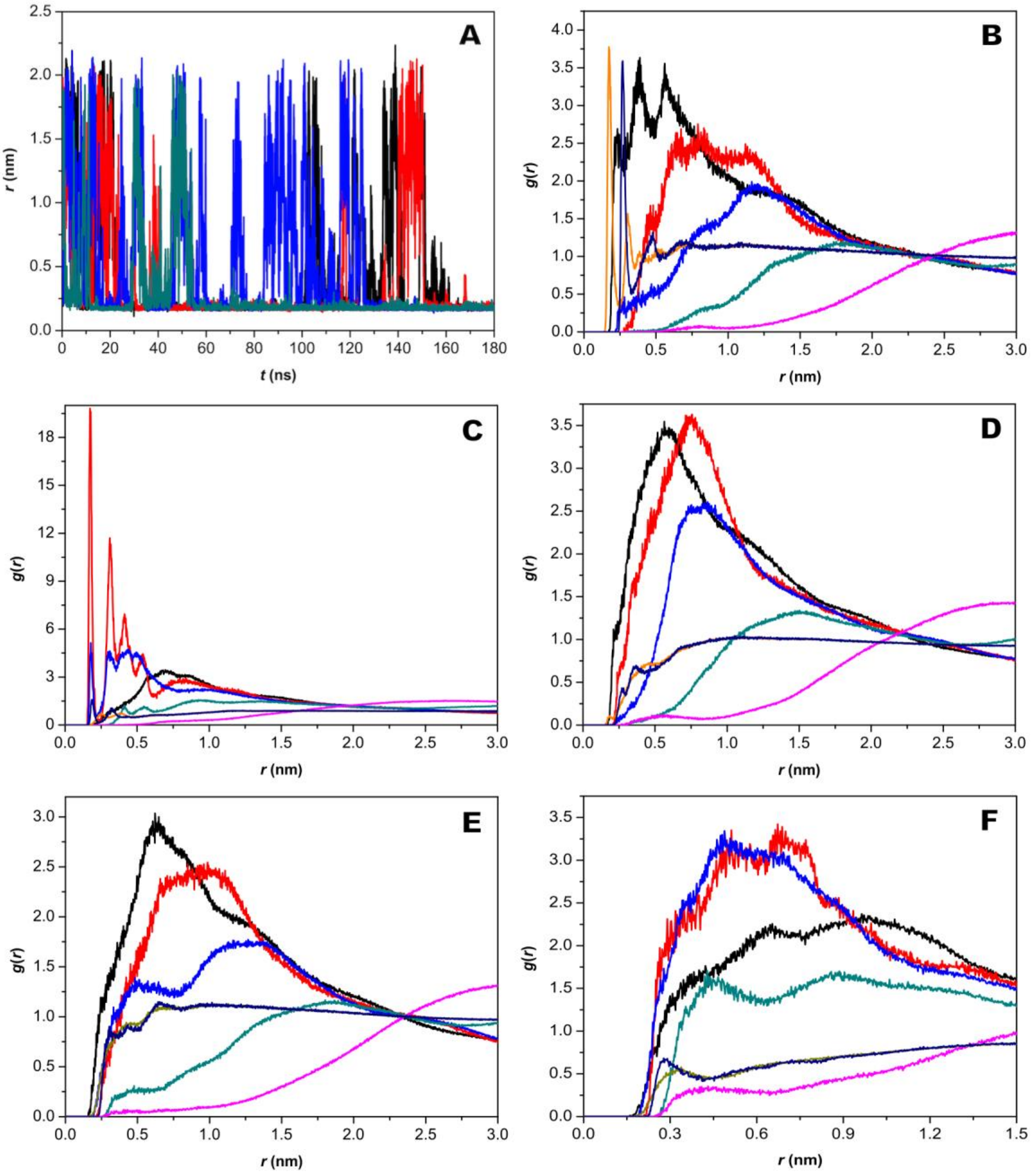


**Figure S3.** (A) Time evolution of the minimum distance between the ampicillin molecules and the POPC bilayer. Each color represents one of the four ampicillin molecules; (B) Radial distribution function (*g*(r)) for distances between ampicillin's carboxylic oxygens and the POPC bilayer system. The colors indicate correlations with the choline group's hydrogen atoms (black), the $PO_4$ atoms (red), the carbon and oxygen's of the ester groups (blue), the carbons 2-10 (cyan) and 11-end (pink) of the aliphatic tail groups, and with the hydrogens (orange) and oxygens (dark blue) of the water molecules. (C) Radial distribution function (*g*(r)) for distances between ampicillin's $NH_3$ hydrogen atoms and the POPC bilayer system. The colors indicate correlations with the choline group's hydrogen atoms (black), the $PO_4$ atoms (red), the carbon and oxygen's of the ester groups (blue), the carbons 2-10 (cyan) and 11-end (pink) of the aliphatic tail groups, and with the hydrogens (orange) and oxygens (dark blue) of the water molecules. (D) Radial distribution function (*g*(r)) for distances between ampicillin's phenylic hydrogen atoms and the POPC bilayer system. The colors indicate correlations with the choline group's hydrogen atoms

(black), the $PO_4$ atoms (red), the carbon and oxygen's of the ester groups (blue), the carbons 2-10 (cyan) and 11-end (pink) of the aliphatic tail groups, and with the hydrogens (orange) and oxygens (dark blue) of the water molecules. (E) Radial distribution function ($g$(r)) for distances between ampicillin's methylic hydrogen atoms and the POPC bilayer system. The colors indicate correlations with the choline group's hydrogen atoms (black), the $PO_4$ atoms (red), the carbon and oxygen's of the ester groups (blue), the carbons 2-10 (cyan) and 11-end (pink) of the aliphatic tail groups, and with the hydrogens (orange) and oxygens (dark blue) of the water molecules. (F) Radial distribution function ($g$(r)) for distances between ampicillin's four membered ring atoms and the POPC bilayer system. The colors indicate correlations with the choline group's hydrogen atoms (black), the $PO_4$ atoms (red), the carbon and oxygen's of the ester groups (blue), the carbons 2-10 (cyan) and 11-end (pink) of the aliphatic tail groups, and with the hydrogens (orange) and oxygens (dark blue) of the water molecules.

## *4. Reaction mechanism of amide bond formation*

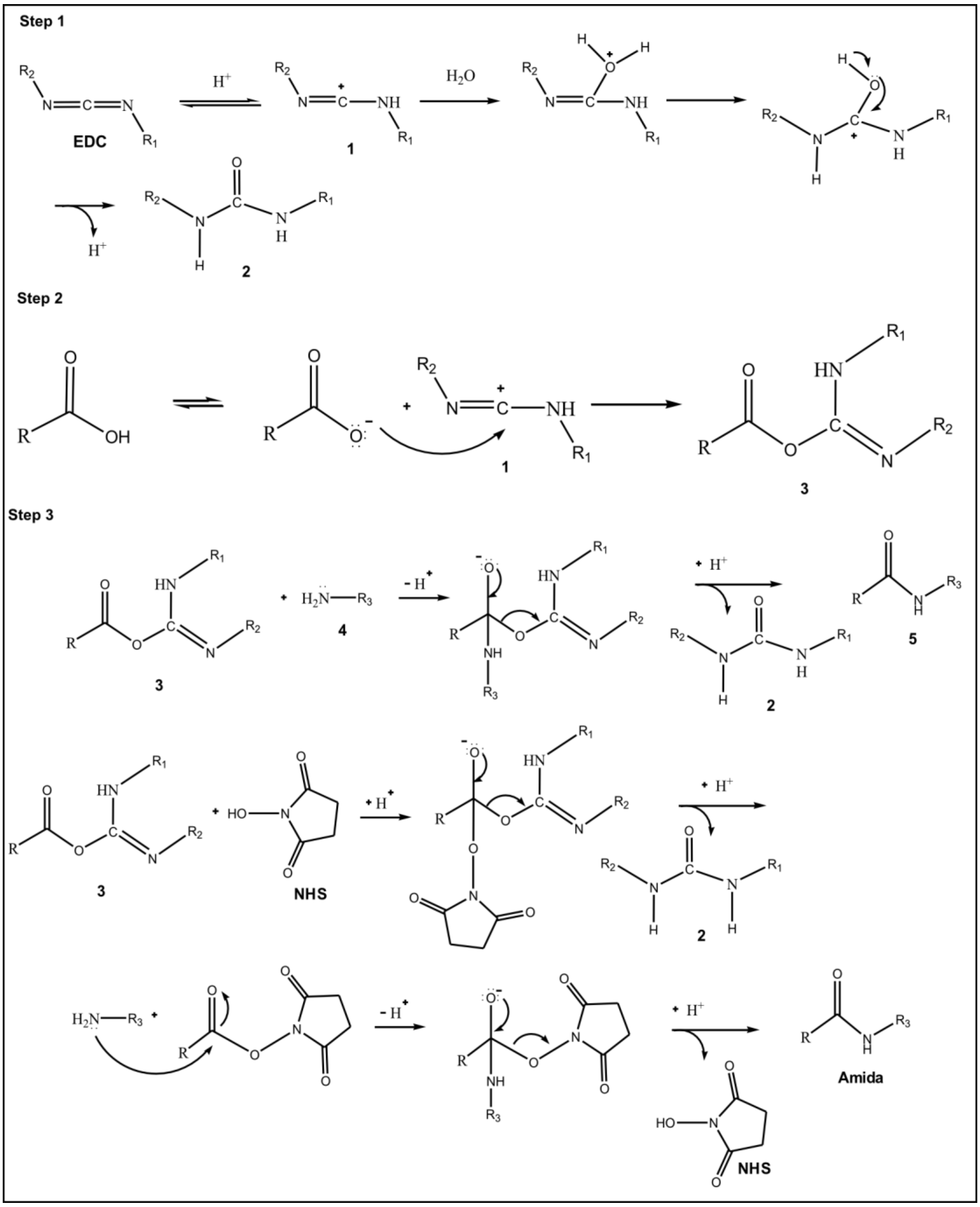


**Figure S4.** Reaction mechanism of amide bond formation. [1]

## 5. FT-IR spectra and TGA curve of synthesized nanoparticles

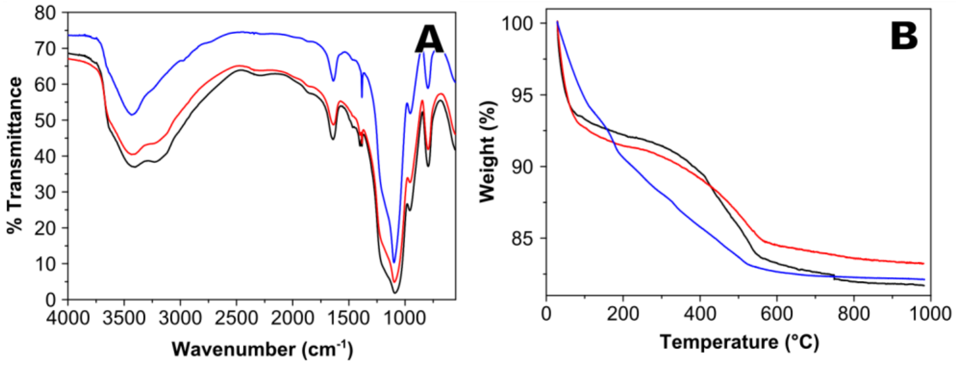


**Figure S5.** (A) Infrared spectrum and (B) TGA curves of synthesized nanoparticles. Black lines: Ag@$SiO_2$; red lines: Ag@$SiO_2$-$NH_2$; and blue lines: Ag@$SiO_2$-Ampicillin.

## 6. Schematic representation of partial pores obstruction during functionalization

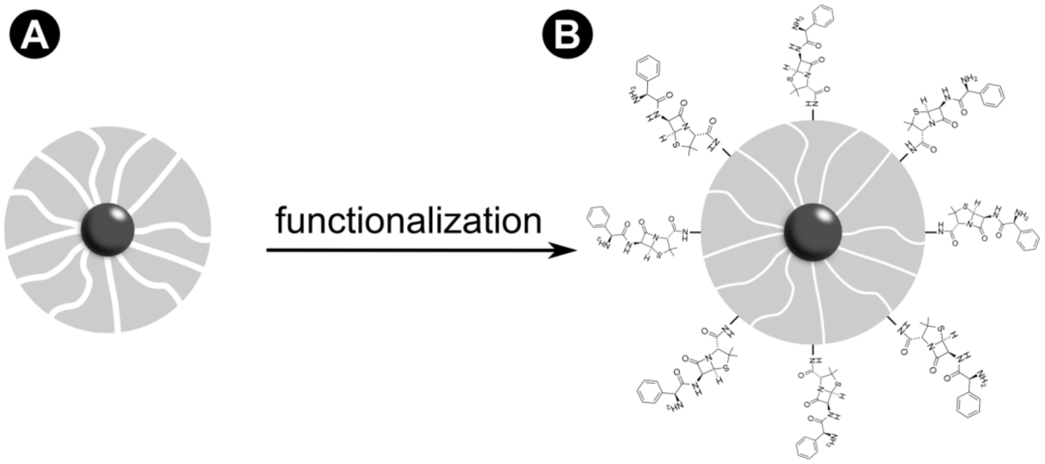


**Figure S6.** Schematic representation of the Ag@$SiO_2$ nanoparticles (A) and Ag@$SiO_2$-Ampicillin (B). The pores in the system Ag@$SiO_2$ are partially obstructed after functionalization process. This scheme is merely illustrative and is not in proportion to the actual size of the system.

### *7. Cytotoxicity assay*

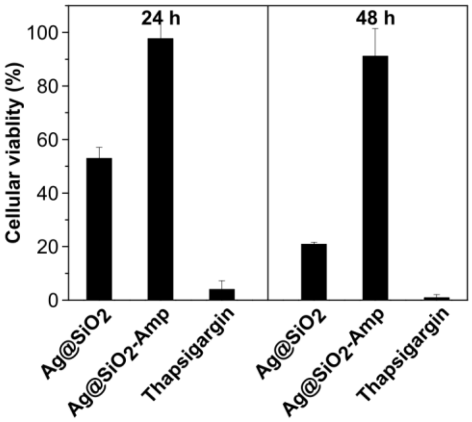


**Figure S7.** Comparative graph of the cytotoxic effect of the synthesized materials to HEK293T cells. Cells were tested by MTS assay after 24 (left) and 48 h (right) of incubation with nanoparticles. Concentration used for Ag@$SiO_2$ and Ag@$SiO_2$-Ampicillin was 372 µg/mL while 30 µM was used for thapsigargin. Data shown are mean for each condition ± SD.

## *8. Penicillin-binding proteins (PBPs)*

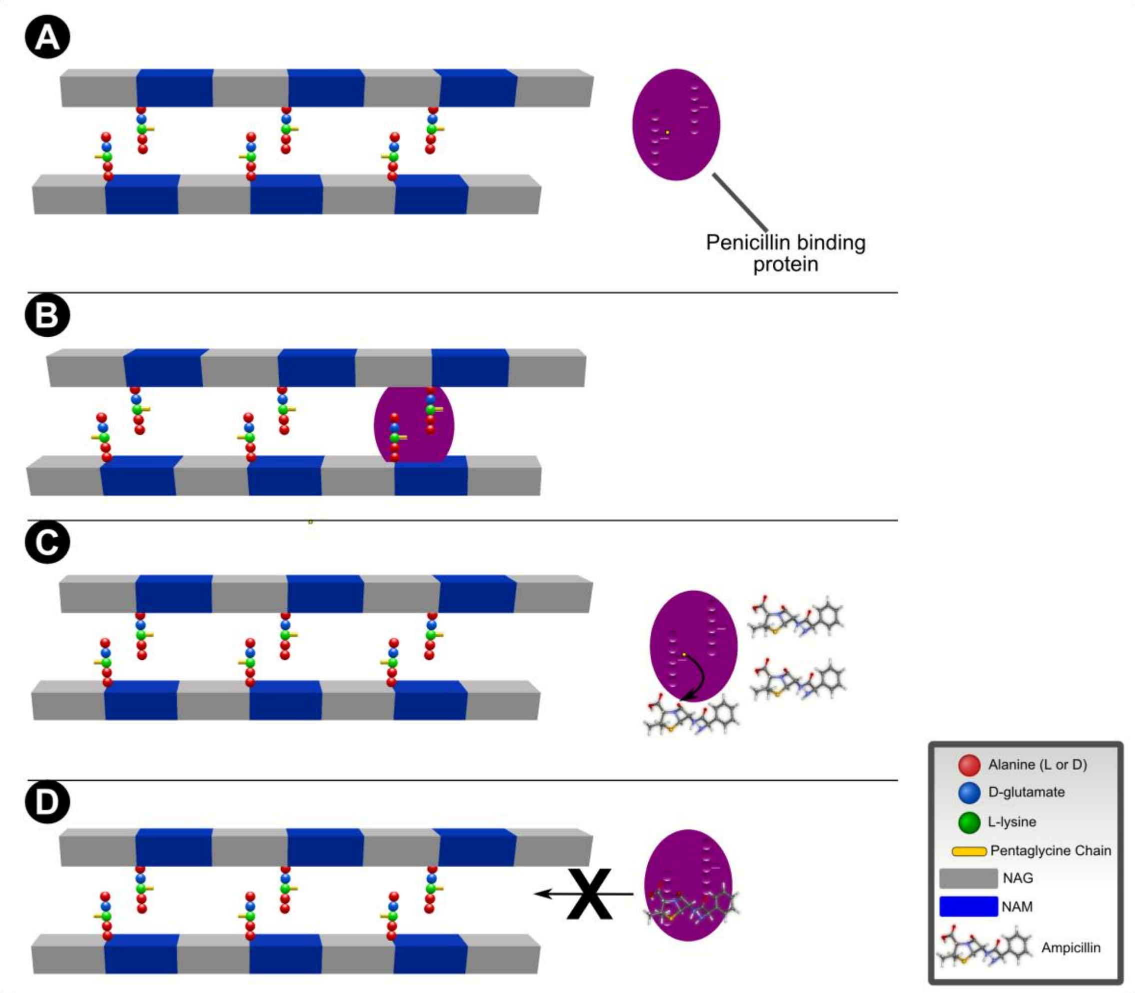


**Figure S8.** PBPs is responsible for catalysis of bacterial cell wall's cross-linking (**Steps A** and **B**). In the presence of penicillin or any other β-lactam antibiotics they can be permanently inhibited (**Steps C** and **D**). (NAM = N-acetylmuramic acid; NAG = N-acetylglucosamine)

## *9. SAXS patterns, size distribution and SEM image for $SiO_2$ nanoparticles*

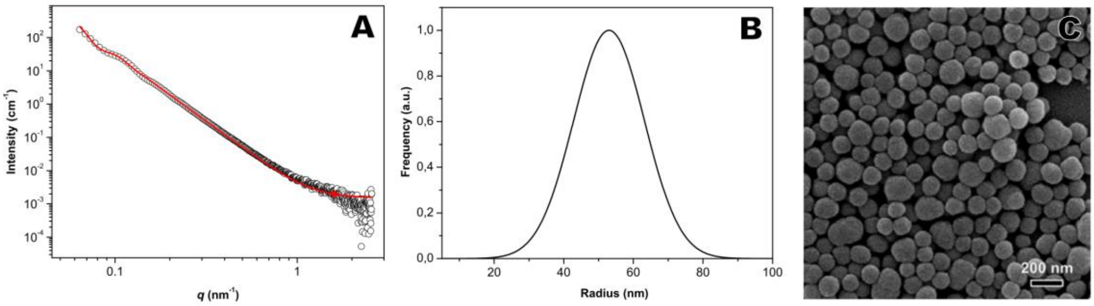


**Figure S9.** (A) SAXS pattern for silica nanoparticles (open circles) and its corresponding fit (solid line). (B) Silica nanoparticles size distribution obtained from SAXS fit presented in Figure S9A. (C) Scanning electronic microscopy of $SiO_2$ nanoparticles.

### *10. Bactericidal tests*

The mass of silver present in the core of Ag@$SiO_2$ was used to normalize the samples concentration and then to calculate the mass of core-shell and silica that should be used during the experiments. The calculations are shown below. Furthermore, based on the TGA results, it was possible to calculate the amount of ampicillin present in the synthesized materials and biological assays with ampicillin were also performed. The concentrations (**a**, **b**, **c**) used in the experiments are presented in **Table S1**.

**Table S1.** Samples concentration (µg/mL) used in biological experiments.

| Sample | [Ag] (µg/mL) | [$SiO_2$] and [$SiO_2$-Ampicillin] (µg/mL) | [Ag@$SiO_2$] and [Ag@$SiO_2$-Ampicillin] (µg/mL) | Ampicillin (µg/mL) |
|---|---|---|---|---|
| **(a)** | 0.10 | 7.33 | 7.43 | 0.15 |
| **(b)** | 1.00 | 73.30 | 74.30 | 1.55 |
| **(c)** | 5.00 | 367.00 | 372.00 | 7.76 |

### *11. Determination of core-shell concentration, using Ag as normalization factor*

According to our TEM results, the silver core has ~14 nm of diameter and the core-shell has ~93 nm. Thus, we first calculate the $SiO_2$ mass present on the core-shell nanoparticle.

$$V_{SiO2} = V_{cs} - V_{core} = (4/3)*\pi*(R_{cs}^3 - R_{core}^3) = (4/3)*\pi*((46.5*10^{-9})^3 - (7*10^{-9})^3)$$
$$= 4.2x10^{-22} m^3$$

Using $d_{SiO2}$ = 2.648 g.cm$^{-3}$ and 1 m$^3$ = 10$^6$ cm$^3$,

$$m_{SiO2} = 4.2x10^{-22} m^3 * (10^6\ cm^3/1\ m^3) * 2.648\ g.cm^{-3} = 1.1x10^{-15} g$$

Then, we calculate Ag mass present on the core-shell nanoparticle.

$$V_{Ag} = V_{core} = (4/3)*\pi*(R_{core}^3) = (4/3)*\pi*((7*10^{-9})^3) = 1.4x10^{-24} m^3$$

Using $d_{Ag}$ = 10.45 g.cm$^{-3}$ and 1 m$^3$ = 10$^6$ cm$^3$,

$$m_{SiO2} = 1.4x10^{-24} m^3 * (10^6\ cm^3/1\ m^3) * 10.45\ g.cm^{-3} = 1.5x10^{-17} g$$

So, to calculate the mass of core-shell needed in biological experiments, we simply use Ag mass as normalization, thus:

| **Core-shell mass** | **Silver mass** |
|---|---|
| **($m_{SiO2}$ + $m_{Ag}$)** | **$m_{Ag}$** |
| ($1.1x10^{-15}$g + $1.5x10^{-17}$g) | $1.5x10^{-17}$g |
| X | Mass of silver needed in the experiment |

We calculate the core-shell mass needed in the experiment, based on the concentration of silver we used in the experiment, therefore 5, 1 and 0.10 µg of silver. The same procedure was used to calculate the mass of $SiO_2$ and $SiO_2$-Amp needed to perform the biological experiments:

| **Core-shell mass** | **Silica mass** |
|---|---|
| **($m_{SiO2}$ + $m_{Ag}$)** | **$m_{SiO2}$** |
| ($1.1x10^{-15}$g + $1.5x10^{-17}$g) | $1.1x10^{-15}$g |
| Mass of core-shell used in the experiment | X |

**Where:**
$V_{SiO2}$ = silica volume
$V_{cs}$ = core-shell volume
$V_{core}$ = core volume

### *12. Synthesis of silica nanoparticles ($SiO_2$)*

Stöber[4] method was used for silica nanoparticles synthesis, which consists of the hydrolysis and condensation of silicon alkoxides. Thus, 400 µL of TEOS were mixed with 4.7 mL of ethanol P.A. under stirring for 5 minutes. Then, 428 µL of $NH_4OH$ were added and left under stirring overnight, at room temperature. The sample was then centrifuged at 8000 rpm for 10 minutes. The supernatant was discarded, the precipitate resuspended in ethanol by centrifuging and then dried at room temperature obtaining nanoparticles of $SiO_2$. Similarly to what described in the Materials and Methods section, silica nanoparticles were functionalized with APTES and ampicillin. The reaction with 3-aminopropyltriethoxysilane (APTES) was performed in two stages using the same reaction flask. Initially, the same procedure previously described for $SiO_2$ nanoparticles synthesis was adopted. After the overnight stirring, 200 µL of APTES were added to the system. The reaction was kept stirred overnight again, followed by centrifugation at 8000 rpm for 10 min to remove excess APTES and TEOS. The precipitate was washed with ethanol and dried to yield the composite $SiO_2$-$NH_2$. The same procedure described in

Materials and Methods was used to obtain $SiO_2$ nanoparticles functionalized with ampicillin ($SiO_2$-Ampicillin).

## References

1. Montalbetti, C. A. G. N.; Falque, V., Amide bond formation and peptide coupling. *Tetrahedron* **2005,** 61, (46), 10827-10852.
2. Nithya Deva Krupa, A.; Raghavan, V., Biosynthesis of Silver Nanoparticles Using Aegle marmelos (Bael) Fruit Extract and Its Application to Prevent Adhesion of Bacteria: A Strategy to Control Microfouling. *Bioinorganic Chemistry and Applications* **2014,** 2014, 949538.
3. Gammoudi, I.; Faye, N. R.; Moroté, F.; Moynet, D.; Grauby-Heywang, C.; Cohen-Bouhacina, T., Characterization of Silica Nanoparticles in Interaction with Escherichia coli Bacteria *World Academy of Science, Engineering and Technology* **2013,** 79, (0), 607-613.
4. Stöber, W.; Fink, A.; Bohn, E., Controlled growth of monodisperse silica spheres in the micron size range. *Journal of Colloid and Interface Science* **1968,** 26, (1), 62-69.
